# Greater exercise tolerance in COPD during acute intermittent compared to continuous shuttle walking protocols: A proof-of-concept study

**DOI:** 10.1177/14799731221142023

**Published:** 2022-12-22

**Authors:** Charikleia Alexiou, Francesca Chambers, Dimitrios Megaritis, Lynsey Wakenshaw, Carlos Echevarria, Ioannis Vogiatzis

**Affiliations:** 1 Faculty of Health and Life Sciences, Department of Sport, Exercise and Rehabilitation, 373117 Northumbria University Newcastle, Newcastle upon Tyne, UK; 2 Pulmonary Rehabilitation Services, 5983 Newcastle upon Tyne Hospitals NHS Foundation Trust, Newcastle upon Tyne, UK; 3 Department of Respiratory Medicine, 5995 Newcastle upon Tyne Hospitals NHS Foundation Trust, Newcastle upon Tyne, UK

**Keywords:** Intermittent exercise, COPD, cardiac output, symptoms

## Abstract

**Objectives:** Ground-based walking is a simple training modality which would suit pulmonary rehabilitation (PR) settings with limited access to specialist equipment. Patients with COPD are, however, unable to walk uninterruptedly at a relatively fast walking pace to optimise training benefits. We compared an intermittent (IntSW) to a continuous (CSW) shuttle walking protocol.

**Methods:** In 14 COPD patients (mean ± SD. FEV_1_: 45 ± 21% predicted) we measured walking distance, cardiac output (CO), arterial oxygen saturation (SpO_2_), and symptoms during (a) an IntSW protocol, consisting of 1-min walking alternating with 1-min rest, and (b) a CSW protocol, both sustained at 85% of predicted VO_2_ peak to the limit of tolerance (Tlim).

**Results:** Median (IQR) distance was greater (*p* = 0.001) during the IntSW protocol (735 (375–1107) m) than the CSW protocol (190 (117–360) m). At iso-distance (distance at Tlim during CSW) the IntSW compared to the CSW protocol was associated with lower CO (8.6 ± 2.6 vs 10.3 ± 3.7 L/min; *p* = 0.013), greater SpO_2_ (92 ± 6% versus 90 ± 7%; *p* = 0.002), and lower symptoms of dyspnoea (2.8 ± 1.3 vs 4.9 ± 1.4; *p* = 0.001) and leg discomfort (2.3 ± 1.7 vs 4.2 ± 2.2; *p* = 0.001). At Tlim symptoms of dyspnoea and leg discomfort did not differ between the IntSW (4.4 ± 1.9 and 3.6 ± 2.1, respectively) and the CSW protocol.

**Conclusions:** The IntSW protocol may provide important clinical benefits during exercise training in the PR settings because it allows greater work outputs compared to the CSW.

## Introduction

Exercise training is the cornerstone of pulmonary rehabilitation (PR) in patients with COPD, principally because it improves exercise capacity, dyspnoea and health-related quality of life (HRQoL).^1^ Despite its clear benefits, PR is grossly underutilised worldwide and is frequently inaccessible to patients; limited resources for PR programmes remains one of the main barriers to its uptake.^2^

Emerging evidence in patients with COPD suggests that PR delivered using minimal equipment leads to clinically important benefits in exercise capacity and HRQoL that are non-inferior to PR delivered using specialist equipment.^3^ Such an approach could potentially increase the geographical coverage and accessibility of PR by expanding the number of settings where PR can be delivered. This is particularly important in low resource countries where access to specialist exercise equipment is scarce.^4^ Given that walking training is simple to perform and easy to administer, there has been growing interest regarding its effectiveness as a training modality to improve exercise capacity and HRQoL in patients with COPD.^5–9^

In the PR setting patients are typically instructed to sustain walking activity at a relatively fast pace ranging between 70 and 80% of the average walking speed assessed during the 6MWT^10^ or between 70 and 85% of predicted VO_2_ peak recorded during the incremental shuttle walking test (ISWT).^5^ However, patients with advanced COPD (i.e. FEV_1_<50% predicted) are potentially unable to sustain a relatively fast walking pace (e.g. 85% of predicted VO_2_ peak) for sufficiently long periods of time to acquire true physiological training effects secondary to intense dyspnoea.^11^ Typically, patients can maintain 5–7 min at 80–85% of predicted VO_2_ peak following the endurance shuttle walking test due to ventilatory and circulatory limitations.^11–14^ It has been suggested that walking pace is reduced to minimise unintended pauses in patients with COPD.^5,15^ Considering that higher intensity training produces greater training benefits, a suboptimal walking pace might compromise the physiological training adaptations in patients with COPD and a faster walking pace with more frequent pauses or slow phases (i.e. intermittent walking) may be preferable.^16^ Indeed, intermittent exercise alternating high intensity work periods with periods of rest or lower intensity exercise is associated with reduced breathlessness and leg discomfort, and greater exercise endurance time and total work output compared to continuous exercise sustained at equivalent work rates.^17–19^ A recently published narrative review^20^ and a systematic review and meta-analysis^21^ suggest that in patients with COPD high intensity intermittent exercise training produces a similar magnitude of change in several physiological outcomes as continuous exercise training. While the aforementioned review studies compared high intensity intermittent to continuous cycling, there are no studies comparing intermittent to continuous walking in patients with COPD.

Accordingly, the primary objective of this study was to compare an intermittent to a continuous shuttle walking protocol, both sustained at 85% of predicted VO_2_ peak in patients with COPD. Based on previous studies in COPD,^17–19^ it was reasoned that intermittent compared to continuous walking would be associated with reduced circulatory and ventilatory loads, and lower breathlessness and leg discomfort, thereby allowing patients to achieve significantly greater walking distance.

## Methods

### Study design

This was a cross-sectional study designed to compare walking distance between a continuous (CSW) protocol and an intermittent (IntSW) protocol in patients with COPD. Consecutive participants were recruited among those enrolled to undertake a PR programme across the Newcastle NHS Trust sites (NuTH) comprising both Royal Victoria Infirmary and Freeman hospitals. On arrival to the site, a respiratory physiotherapist took a medical history (i.e. number of exacerbations during the last 12 months, number of hospitalisation days in the last 12 months, incidence of falls, and comorbidities, such as hypertension, diabetes, heart failure, high cholesterol levels, cancer, arthritis, etc.) and after confirmation of eligibility, patients willing to participate into the study were provided with the necessary information. Upon obtainment of informed consent form patients’ exercise capacity was assessed by the ISWT.

### Study population

Recruitment was performed according to the following inclusion criteria: 1) clinically stable patients with COPD [established by spirometry according to the Global Initiative for chronic lung disease guidelines (GOLD stages II-IV) and forced expiratory volume/forced vital capacity volume ratio (FEV_1_/FVC) < 0.7], 2) male or female COPD patients aged ≥40 years who were referred to NuTH PR programme, 3) optimal medical therapy according to GOLD, 4) current or previous smoking history: ≥10 pack years, 5) able to provide informed consent. Exclusion criteria included: 1) orthopaedic, neurological, or other concomitant diseases that significantly impair normal biomechanical movement patterns, as judged by the investigator, 2) moderate or severe acute exacerbation of COPD within six weeks prior to the study, 3) unstable ischaemic heart disease, including myocardial infarction within 6 weeks prior to the study, 4) moderate or severe aortic stenosis or hypertrophic obstructive cardiomyopathy, 5) uncontrolled hypertension and another condition likely to limit life expectancy to less than 1 year (principally metastatic malignancy).

### Pulmonary function tests

Spirometry was conducted as part of routine clinical care in the Lung Function Laboratory of the Respiratory Medicine Department at the Royal Victoria Infirmary within the past 3 months prior to the study.

### Walking protocols

Experiments were conducted on three visits. On visit one, patients underwent an ISWT to the limit of tolerance (Tlim) according to standardised protocols^22,23^ to establish peak walking distance, symptoms and circulatory responses. Briefly, the ISWT was performed in a course with cones that were 10 m apart. Walking speed was externally paced using a fixed audio tape. “Bleeps” were emitted at regular intervals, at which time patients aimed to be at the opposite end of the course. The ISWT is a progressive maximal test and each minute a triple bleep indicated the increase of walking speed by a small increment (0.17 m/s). Therefore, patients were required to walk faster until they could not keep up with the walking pace. If patients failed to complete a shuttle in the allotted time (>0.5 m away from the cone), the operator terminated the test. No encouragement was given during the performance of the test.

On two separate visits (i.e. visits two and three, separated by a minimum of 48 h) and on the same 10-m course, patients performed two walking protocols to Tlim. Specifically on visit two, patients performed a continuous shuttle walking (CSW) protocol that was sustained at a walking speed corresponding to 85% of the predicted VO_2_ peak derived from the distance walked during the ISWT on visit one according to standardised methodology.^22,23^ Patients were instructed to walk continuously along the course turning around the cones at either end in time with the single bleeps emitted by the audio. Patients were required to walk until they could not keep up with the walking pace. If patients failed to complete a shuttle in the time allowed by the bleeps (>0.5 m away from the cone), the operator terminated the test. No encouragement was given during the performance of the test.

On visit three, patients performed an intermittent shuttle walking (IntSW) protocol consisting of 1-min walking bouts sustained at a walking speed corresponding to 85% of predicted VO_2_ peak derived from the distance walked during the ISWT on visit one, interspersed with 1-min periods of rest. During the 1-min of walking phases, patients were instructed to walk along the course turning around the cones at either end in time with the single bleeps emitted by the audio. At the end of each minute of walking, patients remained standing still next to the cone at either end of the course for 1-min. Patients were required to repeat the 1-min walking bouts until they could not keep up with the walking pace. If patients failed to complete a shuttle in the time allowed by the bleeps (>0.5 m away from the cone), the operator terminated the test. No encouragement was given during the delivery of the test.

Patients using walking aids were allowed to participate in the study. In such cases, the use of walking aids was consistent during all three shuttle walking protocols. In this way patients were able to walk independently and safely.

The CSW protocol was necessarily always performed before the IntSW protocol because endurance time and walking distance were expected to be significantly greater during intermittent than continuous walking. In turn, this was to enable the measurement, during both the IntSW and the CSW protocols of key circulatory variables and symptoms at what we define as iso-distance: i.e. the distance walked during the IntSW protocol corresponding to Tlim of the CSW protocol. Comparing variables at iso-distance between the two modalities will be shown to be critical to the analysis of our data. Ethical approval for this study was obtained (IRAS ID: 280032) and the study was registered in the clinical trials.gov (NCT 04326855).

### Central haemodynamic measurements

During the three tests (i.e. visits, one, two and three), cardiac output (CO), stroke volume (SV) and heart rate (HR) were measured non-invasively and essentially continuously by transthoracic impedance (PhysioFlow PF05; Manatec Biomedical, Macheren, France, PhysioFlow). The cardio-impedance method (PhysioFlow) has previously been validated in patients with COPD for CO measurements against the dye dilution method (invasive method) at rest, across a variety of exercise intensities and at peak exercise (Online supplement).^24–26^

Six electrodes in total were placed according to the manufacturer’s instructions: two (one transmitting and one sensing) on the neck on the left side (one vertically above the other over the carotid artery above the supraclavicular fossa); two (one transmitting and one sensing) anteriorly in the xiphoid region, and another set of electrodes in locations used for conventional single ECG signal monitoring as previously described.^24^ CO values were recorded at 6 s intervals and averaged for offline analysis at 60 s intervals. CO recordings are based on the following equation: CO = HR × SVI × BSA, where CO (litres/minute) is cardiac output, HR (beats/minute) is the heart rate calculated from the time interval of R-Rs determined by the ECG signal, SVI (ml/m^2^) is the stroke volume index per square unit of body surface area and finally BSA (m^2^) is the body surface area expressed in (m^2^), calculated according to the Haycock equation: BSA = 0.024265 × weight^0.53780.5378^ × height^0.39640.3964^.^24^

Systemic oxygen delivery was calculated as the product of CO and arterial oxygen content (CaO_2_); the latter was calculated using the following formula: 1.39 × haemoglobin concentration and fractional arterial oxygen saturation (SpO_2_) measured by a pulse oximeter (Nonin 8600; Nonin Medical, North Plymouth, MN).^27^ Symptoms of dyspnoea and leg discomfort were assessed every minute during the three walking protocols using the modified Borg (1–10) scale.^28^ The locus of walking limitation (dyspnoea, leg discomfort or both, and other reasons) was recorded at Tlim for all three walking tests. Immediately following walking cessation, participants were asked to verbalise their main reasons for stopping and to select qualitative descriptors of their peak exertional dyspnoea. These qualitative descriptors include items related to “unsatisfied inspiration” and “increased work/effort of breathing” within a modified questionnaire, which is presented in Table S1 (Online supplement).^29^

### Statistical analysis

Verification of sample size between the two walking modalities was based on the study by Borel et al.,^30^ using the minimal important difference (MID) estimate of 82 m for the performance of the endurance shuttle walking protocol, a standard deviation (SD) of 113 m, an alpha significance level of 0.05 (2-sided) and 80% power. A minimum sample size of 17 patients was calculated to be sufficient. To compensate for possible dropouts (i.e. 20%)^31^ the sample size was inflated to 20 patients.

Data are reported as means ± SD unless otherwise stated. The Shapiro-Wilk test revealed that all physiological variables and symptoms were normally distributed, whereas walking endurance time and walking distance were not. Comparisons between the CSW and IntSW protocols at Tlim and at iso-distance were made by paired t-tests for all physiological variables and symptoms and by the Wilcoxon signed-rank test for walking endurance time and walking distance. ANOVA with repeated measures was employed to detect significant differences of recorded variables across time between the CSW and the IntSW protocols. When ANOVA detected a significant interaction effect, the Tukey’s post hoc test was used to identify pairwise differences in recorded variables at exact time points of walking between the CSW and the IntSW protocols. In Figures 2 and 3 data are presented at: i) rest, ii) warm up (WU), iii) the first 4 min of walking for which most patients were able to endure during both the CSW and the IntSW protocols and iv) at Tlim for both walking protocols (Tlim_CSW and Tlim_IntSW protocols). Frequency of qualitative descriptors of dyspnoea between walking modalities were analysed by Chi square tests. The level of significance was set at *p* < 0.05.

## Results

### Participant characteristics

**Figure 1.**
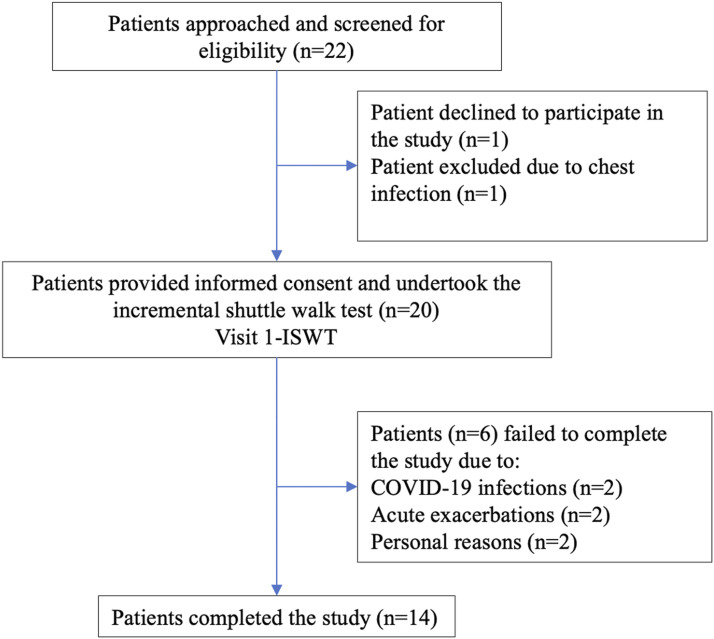
Flow-chart showing participation throughout the study.

Fourteen clinically stable patients with COPD completed all three visits of the study (Figure 1). Patient demographic, anthropometric and lung function characteristics, as well as peak functional capacity data are shown in Tables 1 and 2. Patients exhibited severely impaired lung function (Table 1), reduced peak walking capacity during the ISWT ^32^ with moderate arterial oxygen desaturation (Table 2). Most patients reported dyspnoea as the predominant reason for exercise limitation (Table 2).

**Table 1.** Demographic characteristics.

Sex (M/F)	9/5
Age (years)	66 ± 8
Height (cm)	164 ± 8
Weight (kg)	74 ± 19
BMI (kg/m^2^)	27.75 ± 8.47
FEV_1_ (litres)	1.21 ± 0.71
FEV_1_ (% predicted)	45 ± 21
FVC (litres)	2.54 ± 0.81
FVC (% predicted)	78 ± 22
FEV_1_/FVC (%)	48 ± 18
SBP (mmHg)	138 ± 18
DBP (mmHg)	83 ± 13
mMRC	3 (1)

Values are expressed as mean ± SD or median (IQR) for *n* = 14 COPD patients. SBP, systolic blood pressure; DBP, diastolic blood pressure; mMRC, modified Medical Research Council dyspnoea scale.

**Table 2.** Responses at the limit of tolerance during the ISWT.

ISWT distance (m)	245 ± 128
ISWT (% predicted)	61 ± 31
ISWT time (sec)	296 ± 104
VO_2_ peak predicted (mL/min/kg)	10.32 ± 3.19
CO (L/min)	10.5 ± 3.0
SV (mL/min)	104.7 ± 32.2
HR (beats/min)	102 ± 12
SpO_2_ (%)	91 ± 6
Systemic O_2_ delivery (Litre O_2_/min)	1.92 ± 0.70
Dyspnoea (1-10 Borg score)	4.6 ± 1.3
Leg discomfort (1-10 Borg score)	3.4 ± 2.3
Reason for termination	*n* = 9 due to dyspnoea
	*n* = 2 due to leg discomfort
	*n* = 3 due to dyspnoea and leg discomfort

Values are expressed as mean ± SD for *n* = 14 COPD patients. ISWT, incremental shuttle walking test; VO2 peak, peak oxygen uptake; CO, cardiac output, SV, stroke volume, HR, heart rate, SPO_2_, fractional arterial oxygen saturation.

### Responses between CSW and IntSW protocols

**Figure 2.**
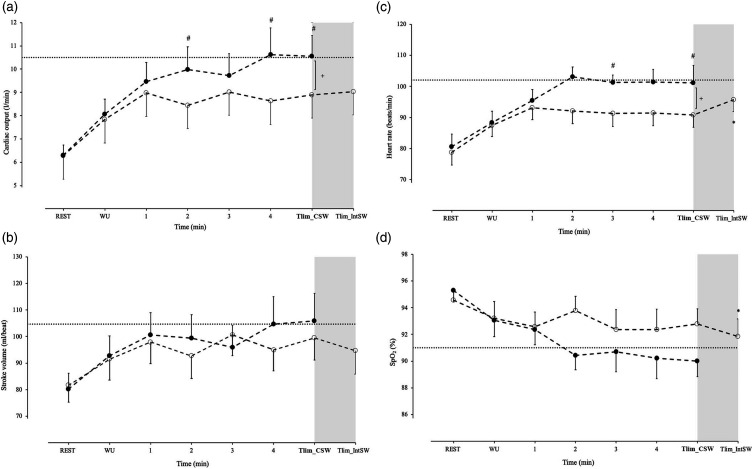
Circulatory responses during a continuous shuttle walking (CSW) protocol and an intermittent shuttle walking (IntSW) protocol: (a), cardiac output, (b), stroke volume, (c), heart rate, (d), arterial oxygen saturation (SpO_2_) recorded at rest, warm up (WU), during the first 4 min of walking and at the limit of tolerance (Tlim_CSW and Tlim_IntSW) for each walking protocol. Values are mean ± SEM for *n* = 14 patients with COPD. + denotes significant difference between CSW and IntSW throughout walking protocols. # denotes significant differences between CSW and IntSW protocols at specific time points of exercise. * denotes significant differences at Tlim between the two modalities. Horizontal dotted lines represent mean responses at Tlim during ISWT.

**Figure 3.**
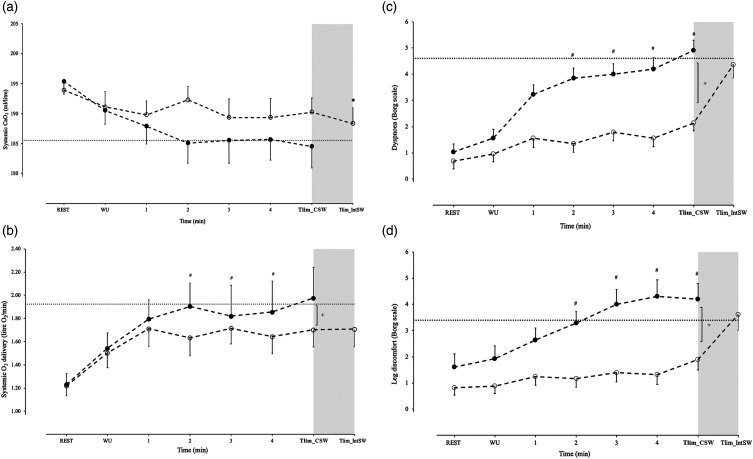
Systemic responses and symptoms during continuous shuttle walking (CSW) and intermittent shuttle walking (IntSW) protocol: (a), systemic arterial oxygen content (CaO_2_), (b), systemic oxygen delivery, (c), dyspnoea, (d), leg discomfort recorded at rest, warm up (WU), during the first 4 min of walking and at the limit of tolerance (Tlim_CSW and Tlim_IntSW) for each walking protocol. Values are mean ± SEM for *n* = 14 patients with COPD. + denotes significant difference between CSW and IntSW throughout walking protocols. # denotes significant differences between CSW and IntSW protocols at specific time points of exercise. * denotes significant differences at Tlim between the two modalities. Horizontal dotted lines represent mean responses at Tlim during ISWT.

During the walking protocols, the CSW protocol compared to IntSW protocol was associated with greater HR (*p* = 0.001), CO (*p* = 0.007), systemic oxygen delivery (*p* = 0.01), symptoms of dyspnoea (*p* = 0.001), and leg discomfort (*p* = 0.03) (Figures 2 and 3). SV, SpO_2_ and CaO_2_ were not different between the CSW and the IntSW protocols (Figures 2 and 3).

### Comparisons at iso-distance

At iso-distance, the IntSW protocol compared to CSW protocol was associated with significantly lower HR (*p* = 0.001), CO (*p* = 0.013), greater SpO_2_ (*p* = 0.002) and lower systemic CaO_2_ (*p* = 0.003), systemic oxygen delivery (*p* = 0.02) and symptoms of dyspnoea and leg discomfort (*p* = 0.001 for both) (Table 3).

**Table 3.** Physiological responses to CSW and IntSW protocols.

	IntSW at Tlim	CSW at Tlim	IntSW at iso-distance
Distance (m)	735 (375; 1107)^a^	190 (117; 360)	179 (128; 317)
Walking time (sec)	1181 (1145; 1740)^a^	203 (152; 355)	N/A
SV (mL/min)	94.7 ± 32.7	100.8 ± 35.7	96.5 ± 31.0
HR (beats/min)	96 ± 14^a^	103 ± 13	91 ± 13^a^
CO (L/min)	9.1 ± 2.8	10.3 ± 3.7	8.6 ± 2.6^a^
SpO_2_ (%)	92 ± 5^a^	90 ± 7	92 ± 6^a^
CaO_2_ (ml/L)	188 ± 100^a^	184 ± 136	189 ± 115^a^
Systemic O_2_ delivery (Litre O_2_/min)	1.70 ± 0.57	1.97 ± 0.75	1.64 ± 0.53^a^
Dyspnoea (1-10 Borg score)	4.4 ± 1.9	4.9 ± 1.4	2.8 ± 1.3^a^
Leg Discomfort (1-10 Borg score)	3.6 ± 2.1	4.2 ± 2.2	2.3 ± 1.7^a^
Dyspnoea as reason for stopping	8	8	N/A
Leg discomfort as reason for stopping	3	5	N/A
Other reason	3	1	N/A
Unsatisfied inspiration	4	8	N/A
Increased work/effort of breathing	11	13	N/A

Values are expressed as mean ± SD or median (IQR) for *n* = 14 COPD patients. SV, stroke volume; HR, heart rate; CO, cardiac output; SpO_2_, fractional arterial oxygen saturation; CaO_2_, systemic arterial oxygen content. Tlim, limit of tolerance; iso-distance, distance walked during the IntSW protocol corresponding to Tlim during the CSW.

^a^significant differences compared to the CSW protocol at Tlim.

### Comparisons at Tlim

At Tlim, walking distance and endurance time was greater for the IntSW protocol (806 ± 572 m and 1481 ± 620 s, respectively) than the CSW (310 ± 288 m and 296 ± 237 s, respectively) (*p* < 0.001 for both) (Table 3). HR, SpO_2_ and CaO_2_ were lower for the IntSW protocol compared to the CSW protocol (*p* ≤ 0.02 for all comparisons) (Table 3). However, at Tlim SV, CO, systemic oxygen delivery and symptoms of dyspnoea and leg discomfort did not differ between the IntSW and the CSW protocols (Table 3 and Figures 2 and 3). At Tlim during the CSW protocol, circulatory responses (CO, HR, and SV), SpO_2_, systemic CaO_2_ and oxygen delivery, as well as symptoms of dyspnoea and leg discomfort reached or exceeded the values recorded at Tlim during the ISWT (Figures 2 and 3). The descriptor of unsatisfied inspiration tended to be twice as frequently reported at Tlim during the CSW protocol compared to the IntSW protocol (*p* = 0.057) (Table 3). There was no difference in the frequency of increased work/effort of breathing between the two modalities (Table 3).

## Discussion

### Summary of main findings

Using our novel intermittent shuttle walk protocol, patients were able to walk for almost four times the distance, and for six times the number of minutes, compared to a conventional continuous shuttle walk protocol. Throughout the walking tests and at iso-distance, circulatory load and symptoms were lower during the IntSW compared to the CSW protocol. However, at the limit of walking tolerance, symptoms of dyspnoea and leg discomfort were not different between the two modalities, suggesting these were important reasons for limiting walking endurance in both modalities.

### Differences in physiological responses between the IntSW and CSW protocols

The IntSW protocol increased walking distance approximately four-fold compared to the CSW protocol. To understand this difference, we can examine the physiological parameters at iso-distance in the IntSW protocol with those at the limit of tolerance during the CSW. We expected that at iso-distance values of recorded variables of importance to walking distance would be more favourable in the IntSW protocol compared to their values at the limit of tolerance in the CSW. From the data presented in Table 3, several factors appear to be involved.

Firstly, a lower degree of arterial hypoxemia at iso-distance during the IntSW protocol suggests that respiratory drive and reliance on anaerobic glycolysis would be lower compared to the CSW protocol (Table 3). This is compatible with reduced sensations of dyspnoea at iso-distance, which could in turn delay the decision to stop exercise (Table 3). The lower likelihood of unsatisfied inspiration (Table 3) at the end of the IntSW protocol suggests that the intermittent protocol gave rest opportunities to recover from breathlessness compared to the CSW protocol. Secondly, cardiac output at iso-distance was lower during the IntSW protocol (Table 3). This leaves some reserve that can be used to prolong walking capacity. In contrast, patients reached their circulatory and oxygen transport limits early on during the CSW protocol (Figures 2 and 3). At the limit of tolerance, symptoms of dyspnoea and leg discomfort were, however, not different between the IntSW and the CSW protocols (Table 3). Therefore, according to this analysis, the likely major reason for terminating exercise in both walking protocols was having reached comparable intensity of symptoms, which took longer during the IntSW protocol compared to the CSW protocol. Whilst the intensity of dyspnoea and the selection frequency of breathing work/effort reflect the awareness of increased motor command output to the respiratory muscles, an increased frequency selection of unsatisfied inspiration has implications for the evolution of the qualitative dimensions of dyspnoea during exercise.^33^ Accordingly, the selection of unsatisfied inspiration in COPD has been associated with the likelihood of increased critical inspiratory mechanical constraints (secondary to dynamic hyperinflation-DH) reflecting the dissociation between increased central neural drive and the capacity to further increase tidal volume.^33^ It is therefore important to appreciate that the likelihood of indicating unsatisfied inspiration was twice more frequent at the limit of tolerance during the CSW protocol compared to IntSW protocol (Table 3). This is consistent with earlier studies showing greater degrees of DH and mechanical constraints to tidal volume expansion during continuous compared to intermittent cycling.^34^

Breathlessness scores at the limit of tolerance of both intermittent and continuous shuttle walking protocols were indicative of moderately severe dyspnoea sensations. Leg discomfort scores were also indicative of moderately severe leg fatigue, thereby suggesting that both sensations constitute limiting factors to walking performance. Accordingly, future studies should employ objective measures to assess peripheral muscle fatigue at the limit of tolerance of walking exercise.^35^

Besides ventilatory constrains limiting walking time and distance during the CSW protocol, circulatory limitation was also apparent, where cardiac output reached equivalent to the ISWT peak levels (Figure 2). This is indicative of reaching minimal circulatory reserve at an early stage during the CSW protocol.

Our findings are consistent with studies in patients with COPD showing that repeated brief bouts of intermittent cycling, followed by equally brief rest periods, are associated with increased cycling tolerance compared to continuous exercise, secondary to lower breathlessness and leg discomfort.^34^ Traditionally, prolonged endurance capacity during intermittent exercise has been attributed to the recovery periods promoting the partial restoration of muscle phosphocreatine levels and the reloading of oxygen myoglobin stores, both of which facilitate a more oxidative degradation of glycogen and thus lower reliance on anaerobic glycolysis during subsequent work periods.^36,37^ Furthermore, our results are consistent with those by Louvaris et al.,^17^ showing that intermittent compared to constant-load cycling at equivalent work outputs is associated with less dyspnoea and leg discomfort and a two-fold increase in endurance cycling time. We may therefore argue that the resting periods incorporated during the IntSW protocol attenuated the reliance on anaerobic glycolysis and thus, the premature occurrence of muscle fatigue secondary to metabolic acidosis.^38^ Moreover, alleviated dyspnoea sensations during the IntSW protocol may be explained by a lower ventilatory demand and rates of DH. Consequently, actual walking endurance time was substantially greater during the IntSW protocol compared to the CSW protocol allowing a four-fold increase in walking distance.

### Implications in the pulmonary rehabilitation setting

In accordance with earlier studies, endurance time during the CSW protocol was limited to approximately 5 minutes.^11,13,23^ The focus of this study was to implement an intermittent walking protocol that could prolong walking distance and endurance time compared to continuous walking rather than introducing an intermittent protocol sustained at peak walking pace. Patients with COPD typically exhibit a lower gait speed compared to their healthy counterparts and experience substantial mobility constraints.^39^ This is the reason why we implemented a sub-maximal, externally paced, shuttle walking speed (equivalent to 85% of predicted VO_2_ peak) across the two walking protocols. Implementation of the IntSW protocol may therefore be well suited to patients with COPD in the PR setting and facilitate physiological adaptations relevant to activities of daily living.

Different ‘real life’ strategies, such as the use of walking aids, have been shown to generally improve functional exercise capacity in patients with COPD.^40^ Evidence suggests that acute use of a rollator during a 6MWT can result in clinically relevant improvements in walking distance (ranging between 19 and 27 m equivalent to 10% of improvement in distance) in parallel to less exertional dyspnoea and increased walking efficiency in patients with moderate to severe COPD.^41^ However, our findings have shown that intermittent walking can increase four times the walking distance compared to continuous walking in patients with similar disease severity. Based on previous studies,^41–43^ a 10% improvement in walking distance is likely to constitute a meaningful improvement. Hence, the four-fold increase in walking distance with intermittent compared to continuous walking may be considered a highly important improvement in walking distance. Indeed, these observations confirm the effectiveness of intermittent walking compared to other strategies, such as downhill walking^44^ in terms of improving exercise endurance capacity and may be particularly important in the design of exercise training programmes in the PR setting.

Moreover, current data emphasise the lack of access to exercise equipment in developing countries or rural and remote areas of developed countries.^45^ From an exercise training perspective, the present study identifies intermittent ground-based walking as a well-tolerated and easy training modality to implement without the need of sophisticated equipment in the community-based PR setting.

### Limitations and future studies

A source of uncertainty in the present study was that the IntSW and CSW protocols were undertaken without the evaluation of ventilatory and gas exchange measurements. This precludes our ability to precisely identify the underlying physiological mechanisms limiting exercise tolerance in both walking tests. For example, lower dyspnoea sensations at iso-distance during the IntSW protocol are indicative of lower ventilatory requirement and degrees of DH. Accordingly, future studies may incorporate measurements of inspiratory capacity, gas exchange and ventilatory variables alongside measurements of arterial blood lactate to better elucidate the physiological limitations of Intermittent walking protocols.

To enable measurements at iso-distance during the intermittent walking protocol (i.e. the distance at exhaustion during the continuous protocol), the continuous walking protocol was always performed before the intermittent walking protocol. This could present a potential limitation of this study. Therefore, we cannot exclude the possibility that there was a learning effect during the intermittent shuttle walking protocol that could have potentially contributed to the four-fold greater distance walked compared to the continuous protocol.

Another note of caution is that patients with COPD experience limited mobility, increased fall risks and reduced balance. To eliminate any gait deficits and unpredictable stride-to-stride fluctuations, patients with COPD use walking aids that might adversely impact on walking pace especially at the end of each shuttle (i.e. turning point) during the walking protocols. Furthermore, the externally paced nature of the shuttle walking testing method compromises the natural control of walking pace.^46^ Taken together, these limiting factors may contribute to early termination of the walking test (>0.5 m away from the cone) and consequently limit the physiological importance of this type of studies.

## Conclusions

Application of intermittent walking in the PR setting may provide important clinical benefits in patients with COPD because it allows greater work outputs with lower symptoms compared to the widely implemented continuous walking protocols.

## Supplemental Material

Supplemental Material - Greater exercise tolerance in COPD during acute intermittent compared to continuous shuttle walking protocols: A proof-of-concept study Click here for additional data file. Supplemental Material for Greater exercise tolerance in COPD during acute intermittent compared to continuous shuttle walking protocols: A proof-of-concept study by Charikleia Alexiou, Francesca Chambers, Dimitrios Megaritis, Lynsey Wakenshaw, Carlos Echevarria and Ioannis Vogiatzis in Chronic Respiratory Disease
