# Supplementary material for: Greater exercise tolerance in COPD during acute intermittent compared to continuous shuttle walking protocols: A proof-of-concept study
Source: Chron Respir Dis. 2022 Dec 22;19:14799731221142023. doi: 10.1177/14799731221142023 (PMC9793067; doi:10.1177/14799731221142023)
Supplement: Supplemental Material - Greater exercise tolerance in COPD during acute intermittent compared to continuous shuttle walking protocols: A proof-of-concept study [file sj-pdf-1-crd-10.1177_14799731221142023.pdf]

Greater exercise tolerance in COPD during acute intermittent compared to continuous shuttle walking protocols: a proof-of-concept study

Online supplement

Assessment of central haemodynamic responses

The cardio-impedance method (PhysioFlow), has previously been validated in patients with COPD for CO measurements against the dye dilution method (invasive method) at rest, across a variety of exercise intensities and at peak exercise (Online supplement).[24-26] Significant correlations (CO: mean difference ~1.0 L/min, 18% during rest and exercise) between the two methods under all conditions examined supported an acceptable agreement of the two methods and the further use of the impedance cardiography based on morphological analysis of the impedance signal during maximal exercise.[24] Similarly, the reliability of the PhysioFlow device has been examined in a series of studies conducted on the cycle ergometer in healthy individuals [26] and patients with respiratory and cardiac diseases [24, 25] during rest and maximal exercise testing. Evidence suggests that impedance cardiography is clinically acceptable for evaluating CO measurements during exercise testing.[24-26]

Bio-impedance cardiography uses the alternations in transthoracic impedance during cardiac ejection to calculate stroke volume values in the process of cardiac output determination.[25] The PhysioFlow device adopts the principle that when emissions of high frequency (75 Hz) and low magnitude (1.8 mA) via electrodes change the current across

thorax during cardiac ejection, stroke volume waveform results can be calculated. By using the Physioflow device, it is not necessary to measure the basal transthoracic impedance and therefore, the location of the electrodes is not critical for the accuracy of the measurements. Additionally, blood resistivity measurements are not needed. [25]

The calibration of the device includes entering patient's demographic characteristics and systolic and diastolic blood pressure values recorded during rest. Initially, SVI is calculated through the calibration phase during rest by evaluating 30 consecutive heart beats during the calibration process when the participant is sitting still and silent according to the following equation: [25] SVical=k x[(dZ/dtmax)/ (Zmax-Zmin)] x W (TFITcal], where K = constant, DZ / dtmax : contractility index, Zmax- Zmin = change in electrical conduction during cardiac contraction, W = algorithm that takes into account blood pressure (systolic-diastolic) as recorded by the sphygmomanometer. TFITcal index is the thoracic flow inversion time measured in the first mathematical derivative of the conductivity signal. This is the time period between the first zero value at the onset of the cardiac cycle (start of the QRS on the ECG) and at the first lower point immediately after the peak of the ejection velocity (dZ / dtmax).

Table S1. Qualitative descriptors of subjective exertional dyspnoea

Qualitative descriptors	Clusters
My breathing requires more work	Increased work
I cannot get enough air in	Unsatisfied inspiration
I cannot take a deep breath in	
My breath does not go in all the way	
Breathing in requires effort	Inspiratory difficulty
My breath does not go in all the way	
Breathing out requires effort	Expiratory difficulty
My breath does not go out all the way	
My chest feels tight	Chest tightness
My chest is constricted	
I feel that my breathing is rapid	Rapid breathing
My breathing feels shallow	Shallow breathing
My breathing is heavy	Heavy breathing
I feel that I am breathing more air	
I feel a hunger for more air	Hunger
I feel that I am suffocating	Suffocation
